# Genetic variations in catechol-O-methyltransferase gene are associated with levodopa response variability in Chinese patients with Parkinson’s disease

**DOI:** 10.1038/s41598-020-65332-2

**Published:** 2020-06-12

**Authors:** Cuiping Zhao, Yihua Wang, Bin Zhang, Yaoxian Yue, Jianyuan Zhang

**Affiliations:** 1 Department of Neurology, Qilu Hospital, Shandong University, Jinan, Shandong China; 2 Department of Neurosurgery, Qilu Hospital, Shandong University, Jinan, Shandong China

**Keywords:** Parkinson's disease, Neurology

## Abstract

Catechol-O-methyltransferase (COMT) is one of the main enzymes in dopamine metabolism and is reported to be associated with susceptibility to Parkinson’s disease (PD) and pharmacotherapy. However, researchers mostly focus on the most common polymorphism, rs4680. In this case-control study, we investigated the association of SNPs other than rs4680 with the levodopa (L-dopa) response and other clinical features in Chinese PD patients. Eleven single nucleotide polymorphisms (SNPs) in the COMT gene were genotyped, and clinical data were collected. Patients with the TT genotype of rs165728 or rs174699 had larger daily levodopa equivalent doses (LEDs) than the patients with CC and CT genotypes under the dominant model (*p* = 0.01421 for rs165728 and *p* = 0.02302 for rs174699). Under the dominant model, the patients with GG at rs4680 G > A had a lower occurrence of dyskinesia than those with AA and AG (*p* = 0.0196). Patients with CC at rs4633 had a lower occurrence of dyskinesia than those with TT and TC (*p* = 0.0429) under the dominant model. The frequencies of the rs174675 T and rs933271 C alleles were higher in PD patients than in the controls (*p* < 0.05). Our primary results showed the possible association of SNPs other than the most common functional rs4680 in COMT with interindividual variance in the L-dopa daily dose and susceptibility to dyskinesia in Chinese patients, although this was an exploratory study based on a small sample size. Larger and more randomized samples are necessary for further investigation.

## Introduction

Parkinson’s disease (PD) is a common progressive age-related neurodegenerative disease with a prevalence from 41/100,000 in people in their 40 s to more than 1900/100,000 in people in their 80s^1^. The main chemical marker of PD is the loss of dopamine production because of the progressive loss of dopaminergic neurons in the substantia nigra in the midbrain^2^. The dopamine precursor levodopa (L-dopa) remains the mainstay of treatment and has been the most effective symptomatic therapy in PD since its introduction^3,4^. In fact, there are considerable inter-individual and intra-individual variations in the response to L-dopa, including motor fluctuations and dyskinesia^3^. “Wearing-off” is a predictable recurrence of symptoms ahead of scheduled doses of dopamine medication, and the symptoms improve after the next dose. Dyskinesia is a kind of involuntary movement from mild to severe and potentially affects 30–40% of PD patients treated with L-dopa. The prevalence of “wearing-off” and dyskinesia was 41–55% over 5 years of treatment with L-dopa. It has been reported that pulsatile stimulation of dopamine receptors accounts for the complications involved in L-dopa therapy.

Nutt JG^5^ reported that 60–90% of the variability in the pharmacokinetics and pharmacodynamics of antiparkinsonian medicine comes from pharmacogenomics. Understanding the pharmacogenetic factors involved in the mechanism, metabolism, transmission and receptors might lead physicians to optimize L-dopa doses and control motor complications^6^. Catechol-O-methyltransferase (COMT) is one of the key enzymes in the metabolism of dopamine, adrenaline and noradrenaline^7^. In terms of the variants involved in L-dopa metabolism, most studies have focused on rs4680 Val158Met in the COMT gene. Hoda^8^ reported the association between the COMT rs4680A > G (Val158Met) polymorphism and PD for the first time in 1996. The activity of COMT with the Val variant is 3- to 4-fold higher than that with the Met variant. The lower COMT enzymatic activity with the Met rather than the Val led to higher prefrontal dopamine availability. Subsequently, an increasing number of studies have been performed to determine the association between the COMT rs4680 polymorphism and PD, including those investigating susceptibility, age of onset and dyskinesia. These studies yielded inconsistent results because of different populations and different recruiting criteria.

Recent findings about the effect of silent mutations in the COMT gene have led us to understand the regulation of the gene not only in exons but also in introns and other untranslated regions. Nackley *et al*.^9^ reported that synonymous single nucleotide polymorphisms (SNPs) could change RNA secondary structure and RNA stability, leading to reduced protein function. There are several studies about the effect of COMT SNPs other than rs4680 on pain sensitivity in cancer patients, the response to different analgesics and the susceptibility to schizophrenia and other behavioural disorders. Few studies have investigated the effect of other SNPs on PD. This study aimed to investigate the relationship between SNPs other than rs4680 in the COMT gene and the variability of the L-dopa response and susceptibility to PD.

## Results

### Characteristics of the study participants

Between March 2015 and March 2016, 73 PD patients met our inclusion criteria, and 77 controls were included. The demographic characteristics of the participants are listed in Table 1. The average age at disease onset was 56.74 ± 10.08 years, and the average duration of the disease was 7.21 ± 2.80 years.

**Table 1:** Demography of PD patients and controls. Means (M) and standard deviations (SD) for the PD patients and controls on background data. PD,Parkinson’s disease.

	PD Patients (n = 73)	Controls (n = 77)
Mean ages(years)	63.95 ± 10.04	54.79 ± 7.48
Age range(years)	43–82	41–78
Female(n)	45	43
Male(n)	28	34
Disease onset(age,years)	56.74 ± 10.08	—
Diseasse duration(years)	7.21 ± 2.80	—

### Genotyping

We selected a total of 11 SNPs and genotyped them in the present study. The details of the SNPs are listed in Table 2. These variants include the missense SNP rs4680 G > A, which has been previously reported frequently in PD and leads to the substitution of valine 158 with methionine, resulting in low COMT enzyme activity, and synonymous rs4633 C > T, which is in the third exon of the COMT gene. rs769224 is synonymous in exon 5; rs4646316 and rs174699 are in intron 5; rs737865, rs4646312, rs933271 and rs174675 are in intron 1; rs2020917 is in the 5′-flanking region; and rs165728 is in the 3′-UTR. SNPs have been reported in other related diseases that involve the COMT gene, such as nicotine dependence, and in cancer patients with pain. The minor allele frequencies (MAFs) of these SNPs (Table 2) were higher than 0.05. There were no significant deviations from Hardy-Weinberg equilibrium (HWE) for the identified SNPs in patients or control subjects (*p* > 0.05).

**Table 2:** Identified SNPs of COMT gene in PD patients and control subjects.

No.	SNP	Chr. Position	SNP Property	Functional Change	Alleles	Minor allele	MAF Patients/control
1	rs4633	19950235	synon_exon 3	p.=(His62His)	C/T	T	0.2123/0.2532
2	rs4646316	19952132	Intron 5	—	C/T	T	0.3904/0.5
3	rs4680	19951271	nonsynon_exon 4	p.Val158Met	G/A	A	0.226/0.2403
4	rs2020917	19928884	5′-flanking	—	C/T	T	0.2945/0.3947
5	rs769224	19951804	synon_exon 5	p.=(Pro199Pro)	G/A	A	0.0959/0.0974
6	rs174675	19934051	Intron 1	—	C/T	T	0.3973/0.2662
7	rs165728	19957023	3′-UTR	—	C/T	C	0.3288/0.3052
8	rs933271	19931407	Intron 1	—	C/T	C	0.4041/0.2662
9	rs174699	19954458	Intron 5	—	C/T	C	0.3288/0.3052
10	rs737865	19930121	Intron 1	—	G/A	G	0.3082/0.4091
11	rs4646312	19948337	Intron 1	—	C/T	C	0.411/0.5

PD, parkinson’s disease; MAF, minor allele frequency; SNP, single nucleotide polymorphism, COMT, Catechol-O- methyltransferase.

### Comparison of frequencies of allele and genotype between PD patients and controls

The allele and genotype frequencies of the identified SNPs in the COMT gene are listed in Table 3. The frequency of the rs174675 T and rs933271 C alleles was significantly higher in PD patients than in controls (for rs174675, *p* = 0.0163, OR [95% CI] = 1.817 (1.116–2.957; for rs933271, *p* = 0.0118, OR [95% CI] = 1.869 (1.149–3.041)).

**Table 3:** Genotype frequencies of the studied SNPs of COMT gene in PD patients and controls.

SNPs	m/M	Additive(patients/controls)				Dominant (patients/controls)				Allele (patients/controls)			
		mm	mM	MM	*p*	mm ± mM	MM	*p*	OR(95%CI)	m	M	*p*	OR(95%CI)
rs174675	T/C	8/3	42/35	23/39	0.0097*^a^	50/38	23/39	0.0182*	2.231(1.146–4.342)	58/41	88/113	0.0163*	1.817(1.116–2.957)
rs933271	C/T	9/3	41/35	23/39	0.0074*^b^	50/38	23/39	0.0182*	2.231(1.146–4.342)	59/41	87/113	0.0118*	1.869(1.149–3.041)
rs737865	G/A	6/11	33/41	34/25	0.0615	39/52	34/25	0.0783	0.5515(0.2843–1.07)	45/63	101/91	0.0696	0.6436(0.3998–1.036)
rs2020917	T/C	6/10	31/40	36/26	0.0639	37/50	36/26	0.0627	0.5344(0.2763–1.034)	43/60	103/92	0.0697	0.6401(0.3953–1.037)
rs165728	C/T	4/7	40/33	29/37	0.6394	44/40	29/37	0.3051	1.403(0.7343–2.682)	48/47	98/107	0.6609	1.115(0.6854–1.814)
rs174699	C/T	5/7	38/33	30/37	0.6451	43/40	30/37	0.3921	1.326(0.695–2.529)	48/47	98/107	0.6609	1.115(0.6854–1.814)
rs4680	A/G	4/5	25/27	44/45	0.7743	29/32	44/45	0.8194	0.9268(0.4829–1.779)	33/37	113/117	0.7708	0.9235(0.5404–1.578)
rs4633	T/C	3/5	25/29	45/43	0.4019	28/34	45/43	0.4712	0.7869(0.4101–1.51)	31/39	115/115	0.4028	0.7949(0.4642–1.361)
rs4646312	C/T	15/21	30/35	28/21	0.1466	45/56	28/21	0.1495	0.6027(0.3027–1.2)	60/77	86/77	0.1223	0.6977(0.4419–1.101)
rs4646316	T/C	13/21	31/35	29/21	0.0720	44/56	29/21	0.1074	0.569(0.2864–1.13)	57/77	89/77	0.0569	0.6404(0.4049–1.013)
rs769224	A/G	0/1	14/13	59/63	0.9641	14/14	59/63	0.8756	1.068(0.4696–2.428)	14/15	132/139	0.9647	0.9828(0.4567–2.115)

*p < 0.05; ^a^*p*_*corrected*_ = 0.0530; ^b^*p*_*corrected*_ = 0.0534;PD,Parkinson’s disease;SNP, single nucleotide polymorphism;COMT, Catechol-O- methyltransferase;CI, confidence interval; M, major allele; m, minor allele; OR, odds ratio.

There were differences in the distribution of genotype frequency between PD patients and controls of SNP rs174675 and rs933271 under the additive model (for rs174675: *p* = 0.0097, OR [95% CI] = 2.078[1.194–3.617], *p*_corrected_ = 0.0530; for rs933271: *p* = 0.00741 OR [95% CI] = 2.115[1.222–3.66], *p*_corrected_ = 0.0534), and there were significant differences in genotype frequency under the dominant model (for rs174675: *p* = 0.0182, OR [95% CI] = 2.231[1.146–4.342]; for rs933271: *p* = 0.0182, OR [95% CI] = 2.231[1.146–4.342]).

### Linkage disequilibrium and haplotype analysis

**Figure 1:**
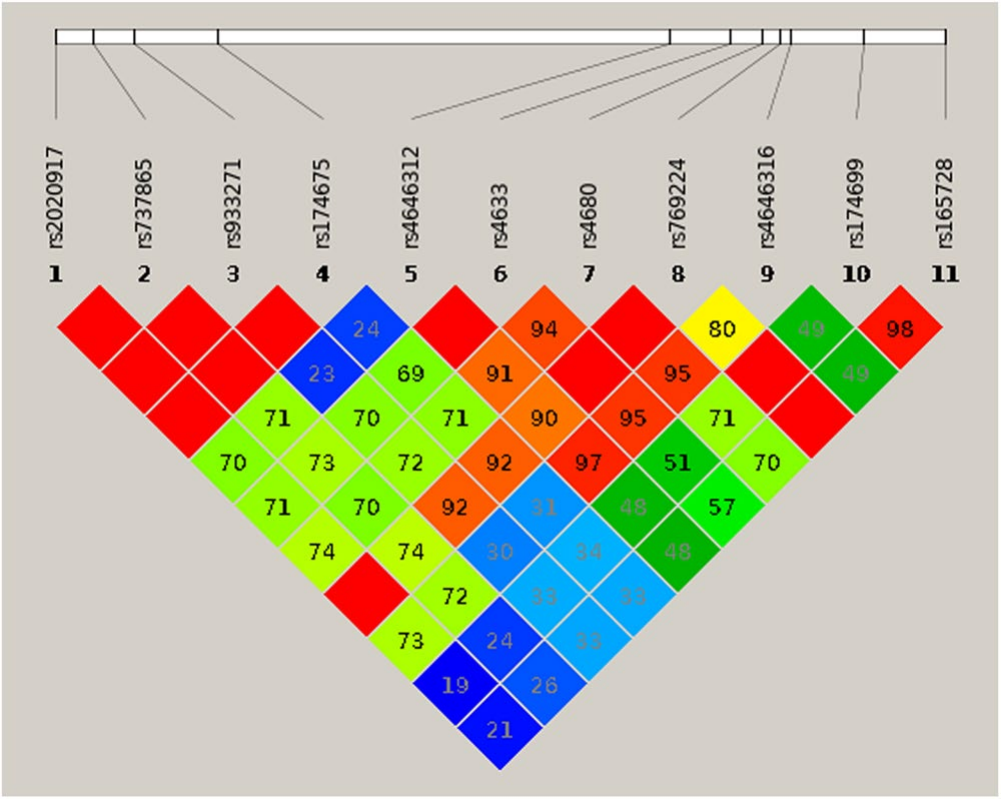
Linkage disequilibrium (LD) pattern of selected 11 SNPs on COMT gene. using the Haploview software. The LD structure indicates the pairwise calculation of D’ for each possible combination of SNPs.

Linkage disequilibrium analysis and haplotype construction for 11 selected COMT SNPs were obtained using Haploview software, and the results are outlined in Fig. 1. The results of the haplotype distribution are shown in Table 4. Linkage disequilibrium analysis showed strong linkage disequilibrium (D’ = 1 or D’ > 0.90) between any two SNPs in each group (group 1: rs2020917-rs737865-rs933271-rs174675, group 2: rs4646312-rs4633-rs4680-rs769224 and group 3: rs174699-rs165728). rs4646316 had strong linkage disequilibrium with rs4646312, rs4633 and rs4680 (D’ > 0.90). Three haplotype blocks were constructed using genotype data for both PD patients and control subjects: COMT1: rs2020917-rs737865-rs933271-rs174675; COMT2: rs4646312-rs4633-rs4680-rs769224; and COMT3: rs174699-rs165728. Four haplotypes derived from COMT1: rs2020917-rs737865-rs933271-rs174675, four derived from COMT-2: rs4646312-rs4633-rs4680-rs769224 and two derived from COMT-3: rs174699-rs165728 were found. The frequency of the “CACT’ haplotype of rs2020917-rs737865-rs933271-rs174675 was higher in PD patients than in controls (OR = 1.925404, 95% CI = 1.071183–3.46083, *p* = 0.028535).

**Table 4:** Haplotype frequencies of COMT gene in PD patients and controls.

Haplotype	Patients (frequency,%)	Controls (frequency,%)	*p*	OR	95%CI
COMT-1: rs2020917,rs737865,rs933271,rs174675					
Hap1-CACT	42.03%	30.60%	0.028535*	1.925404	1.071183–3.46083
Hap2-CATC	33.33%	34.25%	0.868269	0.9564078	0.5648015–1.619535
Hap3-CGTC	2.56%	0.96%	0.414847	2.756757	0.2408756–31.55034
Hap4-TGTC	58.11%	60.78%	—	—	—
COMT-2: rs4646312,rs4633,rs4680,rs769224					
Hap1-CCGA	9.09%	8.93%	0.966539	1.022222	0.3660659–2.854509
Hap2-CCGG	58.14%	64.42%	—	—	—
Hap3-TCGG	35.29%	25.00%	0.060688	1.689092	0.9767348–2.920989
Hap4-TTAG	23.81%	26.06%	0.669445	0.8851418	0.5055238–1.54983
COMT-3: rs174699,rs165728					
Hap1-CC	32.19%	30.52%	0.740065	1.091872	0.6496241–1.835192
Hap2-TT	71.32%	76.43%	—	—	—

*p < 0.05; PD, Parkinson’s disease; COMT, Catechol-O- methyltransferase; CI, confidence interval; OR, odds ratio.

### COMT SNPs and L-dopa response in PD patients

The clinical features of the PD patients with different SNPs are listed in Table 5. For rs165728 and rs174699, there were differences in L-dopa equivalent doses (LEDs) in patients with different genotypes under the additive model (for rs165728: *p* = 0.01144, *p*_*corrected*_ = 0.1113; for rs174699: *p* = 0.02024, *p*_*corrected*_ = 0.1431). Under the dominant model, PD patients with the TT genotype had higher LEDs than patients with the CT and CC genotypes for rs165728 (*p* = 0.01421) and rs174699 (*p* = 0.02302).

**Table 5:** Relationships between clinical features of PD patients and SNPs.

characteristics	rs165728 (n)				rs 174699 (n)				rs4680(n)			
	CC (4)	CT(40)	TT (29)	*p*	CC (5)	CT (38)	TT (30)	*p*	AA (4)	AG (25)	GG (44)	*p*
LED(mg)^a^	481.25 ± 120.85	584.49 ± 156.75	744.19 ± 218.65	0.01144*^c^	505.00 ± 110.34	587.62 ± 160.92	734.38 ± 149.60	0.02024*^d^	537.50 ± 131.25	683.28 ± 194.09	628.50 ± 164.33	0.9011
	**CC** ± **CT** 575.10 ± 134.52		**TT** 744.19 ± 218.65	0.01421*	**CC** ± **CT** 578.01 ± 134.64		**TT** 734.38 ± 149.60	0.02302*	**AA** ± **AG** 663.17 ± 140.14		**GG** 628.50 ± 164.33	0.6223
H&Y^b^	3.0 ± 1.5	3.0 ± 1.0	3.0 ± 1.0	0.8580	3.0 ± 1.5	3.0 ± 1.0	3.0 ± 1.0	0.7310	3.0 ± 2.0	3.0 ± 2.0	3.0 ± 1.0	0.9980
wearing-off(%)	2(50%)	31(77.5%)	21(72.4%)	0.4621	2(40%)	30(78.95%)	22(73.33%)	0.1699	3(75%)	20(80%)	31(70.45%)	0.8135
dyskinesia(%)	3(75%)	13(32.5%)	10(34.48%)	0.3138	3(60%)	13(34.21%)	10(33.33%)	0.5864	1(25%)	14(56%)	11(25%)	0.0363*^e^
	**CC** ± **CT** 16(36.36%)		**TT** 10(34.38%)	0.8694	**CC** ± **CT** 16(37.21%)		**TT** 10(33.33%)	0.7336	**AA** ± **AG** 15 (51.72%)		**GG** 11(25%)	0.0196*
UPDRS-III^a^	37.00 ± 13.65	38.73 ± 8.58	41.68 ± 10.15	0.5457	37.00 ± 12.40	37.80 ± 7.94	42.57 ± 10.82	0.3793	41.50 ± 15.61	37.41 ± 9.47	41.10 ± 8.45	0.7292
UPDRS-IV^b^	4.0 ± 2.0	4.5 ± 2.0	4.0 ± 2.5	0.911	4.0 ± 2.5	5.0 ± 2.0	4.0 ± 2.0	0.781	4.0 ± 2.0	4.0 ± 2.0	5.0 ± 2.5	0.654
UPDRS total^a^	60.00 ± 10.05	71.46 ± 13.14	84.42 ± 12.82	0.09698	60.00 ± 10.05	70.20 ± 11.24	85.35 ± 13.54	0.05901	87.33 ± 23.74	75.13 ± 8.38	75.62 ± 13.72	0.687
MMSE^b^	29.50 ± 1.0	26.0 ± 2.5	26.0 ± 3.5	0.096	29.5 ± 1.0	26.0 ± 2.5	26.5 ± 3.0	0.080	29.0 ± 5.5	25.0 ± 2.5	27.0 ± 3.0	0.116
MoCA^b^	23.00 ± 1.5	21.00 ± 4.5	20.5 ± 4.0	0.590	23.00 ± 1.5	20.0 ± 4.0	21.0 ± 4.0	0.559	26.0 ± 5.0	20.0 ± 3.5	21.0 ± 3.0	0.566
HAMD^b^	14.50 ± 5.0	17.0 ± 5.0	14.5 ± 4.0	0.539	14.50 ± 4.5	17.00 ± 5.0	15.0 ± 5.0	0.839	13.00 ± 6.0	20.0 ± 3.5	15.0 ± 5.5	0.388
HAMA^b^	11.50 ± 6.0	12.5 ± 5.0	7.0 ± 3.5	0.487	11.50 ± 5.0	12.0 ± 4.5	7.0 ± 4.0	0.739	7.00 ± 6.5	12.5 ± 3.5	6.0 ± 4.5	0.238
**characteristics**	**rs4633(n)**				**rs174675(n)**				**rs933271(n)**			
	**TT (3)**	**TC (25)**	**CC (45)**	***p***	**TT (8)**	**CT(42)**	**CC (23)**	***p***	**CC (9)**	**CT(41)**	**TT(23)**	***p***
LED(mg)^a^	579.17 ± 118.97	656.36 ± 121.50	638.66 ± 130.73	0.9853	573.44 ± 109.68	695.71 ± 143.35	568.63 ± 131.59	0.4617	577.78 ± 93.58	697.74 ± 16.11	568.63 ± 93.63	0.4843
H&Y^b^	3.0 ± 1.5	3.0 ± 1.0	3.0 ± 1.0	0.8651	3.0 ± 1.5	3.0 ± 1.0	2.5 ± 1.0	0.1505	3.0 ± 1.0	3.0 ± 1.5	2.5 ± 1.0	0.1143
wearing-off(%)	3(100%)	18(72%)	33(73.3%)	0.7349	5(62.5%)	33(78.6%)	16(69.6%)	0.5377	6(66.7%)	32(78.0%)	16(69.6%)	0.6584
dyskinesia(%)	1(33.33%)	13(52%)	12(26.67%)	0.1051	2(25%)	18(42.86%)	6(26.09%)	0.3223	2(22.22%)	18(43.90%)	6(26.09%)	0.2415
	**TT** ± **TC** 14(48.28%)		**CC** 12(26.67%)	0.0429*	**TT** ± **CT** 20(40%)		**CC** 6(26.09%)	0.2487	**CC** ± **CT** 20(40%)		**TT** 6(26.09%)	0.2487
UPDRS-III^a^	51.00 ± 10.97	36.69 ± ±9.85	40.47 ± 11.20	0.8509	43.00 ± 11.09	42.13 ± 9.54	35.29 ± 10.59	0.2416	44.00 ± 9.69	41.97 ± 11.07	35.29 ± 10.30	0.2261
UPDRS-IV^b^	5.0 ± 3.5	4.0 ± 2.0	5.0 ± 2.5	0.5564	1.5 ± 2.0	5.5 ± 3.0	4.0 ± 2.0	0.8607	3.5 ± 1.5	5.5 ± 3.0	4.0 ± 2.0	0.6552
UPDRS total^a^	87.33 ± 14.66	75.85 ± 17.47	75.29 ± 12.73	0.6182	67.00 ± 11.79	80.37 ± 12.35	69.29 ± 11.42	0.5408	72.75 ± 11.43	80.03 ± 12.81	69.29 ± 9.37	0.4842
MMSE^b^	25.0 ± 5.0	23.5 ± 3.5	25.0 ± 4.0	0.5828	27.5 ± 4.0	24.0 ± 4.0	25.5 ± 3.0	0.6695	27.0 ± 5.0	23.5 ± 4.0	25.5 ± 4.0	0.7413
MoCA^b^	21.5 ± 3.0	18.0 ± 3.5	19.0 ± 4.0	0.7752	22.0 ± 4.0	19.0 ± 3.0	18.5 ± 4.0	0.4387	23.0 ± 4.5	19.0 ± 3.0	18.5 ± 3.0	0.3047
HAMD^b^	13.0 ± 4.0	18.0 ± 3.5	15.0 ± 3.0	0.545	14.5 ± 4.0	17.0 ± 3.5	14.5 ± 4.5	0.5348	12.0 ± 3.0	17.0 ± 2.5	14.5 ± 4.0	0.7867
HAMA^b^	7.0 ± 2.0	12.0 ± 3.0	9.5 ± 3.5	0.6548	13.5 ± 3.0	11.0 ± 3.5	8.5 ± 3.0	0.2904	9.0 ± 2.0	11.0 ± 3.5	8.5 ± 4.0	0.5354

*p < 0.05; ^a^Values are expressed as mean ± SD; ^b^Values are expressed as median ±IQ.^e^*p*_*corrected*_ = 0.1113, ^d^*p*_*corrected*_ = 0.1431, ^e^*p*_*corrected*_ = 0.2857; LED, L-dopa equivalent doses; H&Y, Hoehn and Yahr stage; UPDRS, Unified Parkinson’s Disease Rating Scale; MMSE, mini-mental state examination; MoCA, Montreal Cognitive Assessment; HAMD, the Hamilton Depression Scale; HAMA, the Hamilton Anxiety Scale.

There were differences in the occurrence of dyskinesia among PD patients with different genotypes of rs4680 G > A under the additive model (*p* = 0.0363, *p*_*corrected*_ = *0.2857*). Under the dominant model, the patients with GG at rs4680 had a lower occurrence of dyskinesia than those with AA and AG (*p* = 0.0196). Patients with CC at rs4633 had a lower occurrence of dyskinesia than those with TT and TC (*p* = 0.0429) under the dominant model. No significant difference was observed in other clinical features, including H&Y stage, UPDRS-III, UPDRS-IV, UPDRS total score, HAMA, HAMD, MMSE or MoCA, with the SNPs above. For other SNPs and derived haplotypes, there were no significant differences in any studied clinical features.

## Discussion

Although PD is a common degenerative disease and L-dopa is the most effective medicine for relieving symptoms, the cause of considerable inter-individual variations in response to L-dopa is not very clear. Variations in COMT activity might be associated with these variations in response to L-dopa in PD patients because COMT is one of the most important enzymes involved in dopamine metabolism. Here, we investigated the association between several COMT SNPs in addition to functional rs4680 and the L-dopa response in PD. We found that patients with the rs165728 TT and rs174699 TT genotypes needed larger daily LEDs than patients with the CT and CC genotypes under dominant models. It was supposed that the substitution of the T with the C allele lowers COMT enzymatic activity, leading to a decreased dose of L-dopa. Though rs165728 is located in the 3’-UTR and rs174699 is located in intron 5, they possibly alter RNA secondary structure, affecting RNA stability and leading to decreased COMT activity, but the mechanism needs further study. Several published studies have focused on the association of the polymorphism of COMT with L-dopa treatment^10–13^. Xiao Q^11^ reported that the T allele of rs4633, A allele of rs4680 or TT/AA alleles of rs4633–4680 had larger daily LEDs. In our primary study, rs4680 G > A was not associated with the LED. Yin B^10^ found that allele C of rs4633 was associated with the severity of PD but not with L-dopa medication. Contin M^12^ failed to identify a relevant L-dopa pharmacokinetic–pharmacodynamic response associated with rs4680 G > A by measuring plasma L-dopa concentrations after a fasting oral L-dopa test with L-dopa (100 mg)/benserazide (25 mg). Bialecka M^13^ found that LEDs (at the fifth year of PD) for G_C_G_G haplotype carriers (rs6269-rs4633-rs4818-rs4680) were significantly higher than those for non-carriers. There were inconsistent results, probably because of the different study protocols and populations used. The disease duration of the patients in the study of Yin B and Xiao Q showed great discrepancy (PD duration of 1–14 years^10^ with a mean duration of 6.0 ± 5.0 years^11^). Our primary results were similar to those of of Bialecka M^13^, and we calculated and compared the daily LEDs at approximately the fifth year for PD patients with different SNPs.

Epidemiological evidence has demonstrated that the occurrence of dyskinesia and wearing-off varies significantly. Many patients will never develop motor complications. These studies suggest that endogenous factors may play an important role in individual complication susceptibility despite well-known risk factors, including younger age of onset, greater severity, longer disease duration of PD and higher LED^14^. Recently, a meta-analysis^15^, which included a total of five studies, showed that allele A of rs4680 was correlated with wearing-off risk in PD. One study^16^ found that the rs4680 G allele might be a risk factor for the “wearing-off” phenomenon in 1087 Chinese PD patients. However, the average disease duration in the study was 3 ± 5 years, and only 15.1% of patients exhibited wearing off. In a study by Xiao Q^11^, though the mean disease duration was 6.0 years, there was great discrepancy in duration among patients (SD = 4.0 years), and only 23.1% of patients demonstrated wearing-off. We failed to determine the association between polymorphism of COMT and wearing-off. As many as 73.97% of the PD patients in our study exhibited wearing off because we included patients with a relatively long disease duration (5–10 years) to decrease the effect of the discrepancy in disease duration. This implied that other factors, such as duration and severity, had a greater impact on wearing off than genetic effects.

In addition to the wearing-off phenomenon, dyskinesia is another common dopamine-related motor complication. Lonneke M.L^17^. reported that the A-allele of the COMT Val158Met polymorphism was related to an increased risk of developing dyskinesias during follow-up. In his cohort, 45% of the PD patients developed dyskinesia. In the Xiao Q ^11^ study, the authors failed to arrive at the same conclusion because a limited number of patients (only 12.6%) presented with dyskinesia. Recently, Ivanova SA^18^ found an association between four SNPs—rs165774, rs4818, rs4633, and rs4680—and L-dopa-induced dyskinesia. However, the results did not reach statistical significance when the duration of disease was added as a covariate in regression analysis, and only the additive model for rs165774 was found to be close to statistical significance. In their cohort, the mean disease duration was 9.8 ± 5.6 years, and 25% of patients developed dyskinesia. In our cohort, 35.74% developed dyskinesia, and the mean duration was 7.21 ± 2.80 years. We included patients with a disease duration of more than 5 years because most of the suspected patients would have motor complications after a duration of 5 years, which is usually considered a landmark. Our primary study showed that patients with GG at rs4680 had a lower occurrence of dyskinesia than those with AA and AG. Patients with CC at rs4633 had a lower occurrence of dyskinesia than those with TT and TC. There were no conclusive results about the relationship of SNPs with dyskinesia, possibly because of the great difference in the inclusion criteria. Further studies with larger samples and stricter disease duration limitations are needed.

There is significant heterogeneity in COMT gene variants in PD in different ethnic groups. A meta-analysis^19^ that included 24 studies for the COMT rs4680 SNP in 9719 PD patients and 14634 controls showed that the COMT rs4680 polymorphism was not a major risk determinant for PD. Recently, Yan-chun Wang^20^ pooled 27 studies including 10239 PD patients in their meta-analysis and found a significantly closer association between the COMT Val158Met polymorphism and PD in Japanese and Indian populations than in other ethnicities. Similar results were obtained in Lixue Chuan’s study showing that the Val158Met polymorphism was an associated risk factor for PD in Asian rather than Caucasian populations and further found that it might be associated with PD in Japanese rather than Chinese populations^21^. Xiao Q^11^ from China reported that rs4633 and rs4680 polymorphisms were associated with susceptibility to early onset PD (EOPD, onset before 50 years of age) but not late onset PD (LOPD, onset after 50 years of age) or in general. However, there were only 24 EOPD patients, and larger cohort studies on EOPD patients are still needed in the future. Similarly, our primary study failed to identify an association of missense SNP rs4680, synonymous SNP rs4633 and rs769224 with susceptibility to PD, but a larger sample size is needed for verification. We found that the frequencies of the rs174675 T and rs933271 C alleles were higher in PD patients than in the controls. These two SNPs were studied in nicotine dependency^22^ but have never been studied in PD before. Further studies with larger samples are still needed to verify these two SNPs.

Our primary and exploratory study had several limitations. The present study aimed to investigate the association of SNPs other than rs4680 of the COMT gene with medication response variability and susceptibility to PD based on a small sample size. Larger and more randomized samples are necessary to investigate the relationship between SNPs rs174675 T, rs933271 C, rs4680 A, and rs4633 T and the susceptibility of PD. This requires a very large sample size of thousands of PD patients according to the calculation of statistical power. However, our present study had stricter inclusion criteria on onset age and disease duration, in order to decrease the discrepancy of the effects of onset age and duration on clinical features. In future studies, we plan to carry out two isolated research protocols. One is to assess the relationship between SNPs and the risk of PD with a more randomized, larger sample, and the other is to investigate the relationship between SNPs and clinical features with stricter inclusion criteria.

## Conclusions

In conclusion, we presented here the possible association of SNPs other than the most common functional rs4680 in COMT with interindividual variance in LEDs, susceptibility to dyskinesia and the risk of PD in Chinese patients, which may be useful in leading physicians to optimize individualized dopaminergic medicine for patients with PD.

## Methods

### Samples

We recruited idiopathic PD patients from the movement disorder clinic at the Department of Neurology of Qilu Hospital (Qingdao), Shandong University, and age- and gender-matched healthy individuals as the control group during the period from March 2015 to March 2016. All patients with PD who were eligible for this study were diagnosed with idiopathic PD according to the UK Brain Bank criteria^23^. None of these patients reported a family history of neurodegenerative disorders or Parkinson’s disease. The disease duration of all included patients was nearly or more than 5 years to have high accuracy of diagnosis and comparability of phenotype. All individuals who were diagnosed with atypical parkinsonism, including multiple system atrophy (MSA), progressive supranuclear palsy (PSP), vascular or drug-induced parkinsonism, Alzheimer’s disease (AD), frontotemporal dementia (FTD) or dementia with Lewy body (DLB), were excluded from our study. Control subjects had extensive neurological and medical examinations, which showed that they were free of significant illness and neurodegenerative disease. These participants were unrelated Han Chinese in origin. Each participant was given oral and written information about the purpose of the study and signed the informed consent form. The study was performed in accordance with The Code of Ethics of the World Medical Association (Declaration of Helsinki) for experiments involving humans, and the protocol was approved by the Research Ethics Committee of Qilu Hospital affiliated with Shandong University.

### Genotyping

Genomic DNA was extracted from peripheral blood samples collected from each individual using the standardized phenol/chloroform extraction method. The selection of SNPs for this study was based upon the frequency of SNPs and the position in the gene (HapMap Public Release #27 CHB, version of 2009–02–06), and what was known in the literature at the time of the research was planned. Eleven SNPs were genotyped in the present study, and the details are listed in Table 2. Primers were designed by Primer 3 software (http://frodo.wi.mit.edu/cgi-bin/primer3/ primer3_ www.cgi).

The selected 11 SNPs were analysed in the PD and control groups by polymerase chain reaction-ligase detection reaction (PCR-LDR) on an ABI Prism 377 Sequence Detection System (Applied Biosystems, Foster City, CA) technically supported by the Center for Human Genetics Research, Shanghai Genesky Biotechnology Company. GeneMapper Software v4.0 (Applied Biosystems) was used for data analysis. Haplotype blocks were estimated following the default procedure in Haploview.

### Data collection

In this study, clinical data were collected through face-to-face interviews and questionnaires assessing the clinical characteristics of patients with PD that were conducted by at least two movement disorder specialists. PD clinical characteristics included disease duration, age of onset, medicine and motor complications. All patients were examined during the “on” state using the Movement Disorder Society-Unified Parkinson’s Disease Rating Scale (MDS-UPDRS), and disease severity was evaluated using the classification of Hoehn and Yahr stage (H&Y stage). The L-dopa equivalent doses (LEDs) at approximately 5 years (as close to the 5th year as possible, average 6.353 ± 1.89) of disease duration were calculated using a method reported in a previous study^24^. The PD-related non-motor symptoms were evaluated using the Mini-Mental State Examination (MMSE), Montreal Cognitive Assessment (MoCA), Hamilton Anxiety Scale (HAMA) and Hamilton Depression Scale (HAMD).

### Statistical analysis

In descriptive analyses, mean and standard deviation (mean ± SD) were used for normally distributed continuous variables, median and interquartile range (median ±IQ) for continuous variables with skewed distribution, and proportions for categorical variables. PLINK software was used to test HWE to compare the allele frequency between PD patients and controls. Chi-square analysis, Fisher’s exact test or logistic regression was used to compare the COMT allele frequencies or genotype frequencies between the control group and PD group under the additive, dominant, and recessive genetic modes. The false discovery rate (FDR_BH) adjusted for the *p*-value was used for correcting multiple tests.

Analysis of variance of factorial design was used to perform the association between COMT polymorphisms and clinical outcomes, adjusted for age and sex under the additive, dominant, and recessive genetic modes. A binary logistic regression model was used to analyse the relation between SNPs or haplotypes and clinical phenotype with age and sex as covariates. All of the above statistical analyses were carried out using SPSS 19 statistical software(SPSS Inc., Chicago, IL). Haplotypes and linkage disequilibrium (LD) measurements were implemented within the Haploview 4.2 program. All statistical significance was defined as *p* < 0.05.
